# MicroRNA-26a/cyclin-dependent kinase 5 axis controls proliferation, apoptosis and *in vivo* tumor growth of diffuse large B-cell lymphoma cell lines

**DOI:** 10.1038/cddis.2017.291

**Published:** 2017-06-22

**Authors:** Floriana Maria Farina, Alessandra Inguscio, Paolo Kunderfranco, Alice Cortesi, Leonardo Elia, Manuela Quintavalle

**Affiliations:** 1 Humanitas Clinical and Research Center, Rozzano, Italy; 2 Department of Medical Biotechnology and Translational Medicine, University of Milano, Milano, Italy; 3 INGM, Milan, Italy; 4 Department of Molecular and Translational Medicine, University of Brescia, Brescia, Italy

## Abstract

Diffuse large B-cell lymphoma (DLBCL) is the most frequent type of non-Hodgkin lymphoma. Despite a favorable therapeutic response to first-line chemo-immunotherapy, still 30–40% of patients is refractory, or relapse after this treatment. Thus, alternative strategies must be sought. Previous studies have indicated that cyclin-dependent kinase 5 (CDK5), a serine/threonine protein kinase, is involved in tumor development and progression, and it may represent a potential therapeutic target. However, its role in modulating DLBCL growth and progression remains largely unexplored. In this study, we show that CDK5 and its activator, cyclin-dependent kinase 5 activator 1 (CDK5R1 or p35), are overexpressed in DLBCL cell lines and that signal transducer and activator of transcription 3 (STAT3) phosphorylation and activity is dependent on CDK5 expression in DLBCL. Using public data sets, we also demonstrate that patients with DLBCL show a higher expression of CDK5 compared with healthy individuals. By using loss-of-function approaches, we demonstrate that CDK5’s activity regulates proliferation and survival of DLBCL cells. MicroRNAs (miRNAs or miRs) are small noncoding RNAs that negatively regulating gene expression and are involved in cancer initiation and progression. We identify miR-26a as direct regulator of p35 expression and CDK5 activity. We show that miR-26a expression is lower in DLBCL cell lines compared to B lymphocytes and that its ectopic expression leads to a drastic reduction of DLBCL tumor growth *in vivo* and decreased proliferation, cell-cycle progression, and survival *in vitro*. Remarkably, concomitant overexpression of a 3′-UTR-truncated form of p35 promoted tumor growth *in vivo* and cell proliferation, cell-cycle progression, and cell survival *in vitro*. In conclusion, these results demonstrate an important role for miR-26a and CDK5 together in the survival and growth of DLBCL cells, suggesting the existence of potential novel therapeutic targets for the treatment of DLBCL.

B-cell non-Hodgkin lymphomas (NHLs) are a group of lymphoproliferative B-cell disorders that include, among others, diffuse large B-cell lymphoma (DLBCL), the most frequent type of NHL.^1^ Although the immunochemotherapy-based regimen R-CHOP (rituxan, cyclophosphamide, oncovin, prednisone) represents a curative approach for an average 50% of DLBCL patients, a substantial proportion of patients (30–40%) shows refractory disease or early relapses (<12 months) after standard regimens. Chemorefractory DLBCL, undergoing second-line salvage treatment, have a poor long-term disease control (3-year progression-free survival of ~20%), even after high-dose chemotherapy and autologous stem-cell transplantation.^2^ Therefore, identifying alternative therapeutic targets is of utmost importance.

CDK5 was initially identified for its homology with the other cyclin-dependent kinases. However, it was later demonstrated that CDK5 is not activated through binding with cyclins, rather with two other proteins, p35 and p39, or their cleaved forms, p25 and p29. While no clear role for CDK5 in cell cycle regulation has yet been shown, CDK5 has been described to have an important role in cell migration, cell adhesion, myogenesis, apoptosis, and senescence (all reviewed in).^3^ Moreover, CDK5 has been described to play a significant role in many malignancies such as prostate cancer,^4^ pancreatic cancer^5^ and glioblastoma,^6^ and to be a regulator of cancer invasion and metastasis by influencing the promotion of invasive structures called invadopodia.^7^ Despite progress in understanding the roles of CDK5 in human cancer, the involvement of CDK5 in the proliferation and apoptosis of DLBCL cancer cells is yet uninvestigated.

In recent years, microRNAs (miRNAs or miRs) have emerged as important players in cancer progression. They are involved in many cellular processes such as apoptosis, proliferation, and differentiation,^8^ and can function either as tumor suppressors or oncogenes.^9^ MiR-26a, together with miR-26b, belongs to the miR-26 family. The role of miR-26a in cancer cells is controversial. On the one hand, miR-26a has been demonstrated to be downregulated and to act as tumor suppressor in breast cancer,^10^ nasopharyngeal carcinoma,^11^ hepatocellular carcinoma,^12^ and gastric cancer.^13^ On the other hand, it has been described to act as an oncogene in glioma^14^ and cholangiocarcinoma.^15^ Until now, the role of miR-26 in DLBCL was undefined.

In this study, we investigated the involvement of miR-26a and CDK5/p35 in DLBCL. We examined the expression level of CDK5/p35 and miR-26a in human DLBCL cell lines and tested loss- and gain-of-function effects on cell growth and apoptosis. We also investigated the role of miR-26a on DLBCL tumorigenesis in a murine model. Our study indicates that p35 deregulation through miR-26a plays an important role in DLBCL growth and, following further studies in patient samples, it may be considered as a novel prognostic marker and a potential therapeutic target for DLBCL.

## Results

### CDK5 is over-activated in lymphoma cell lines and over-expressed in DLBCL patients

**Figure 1:**
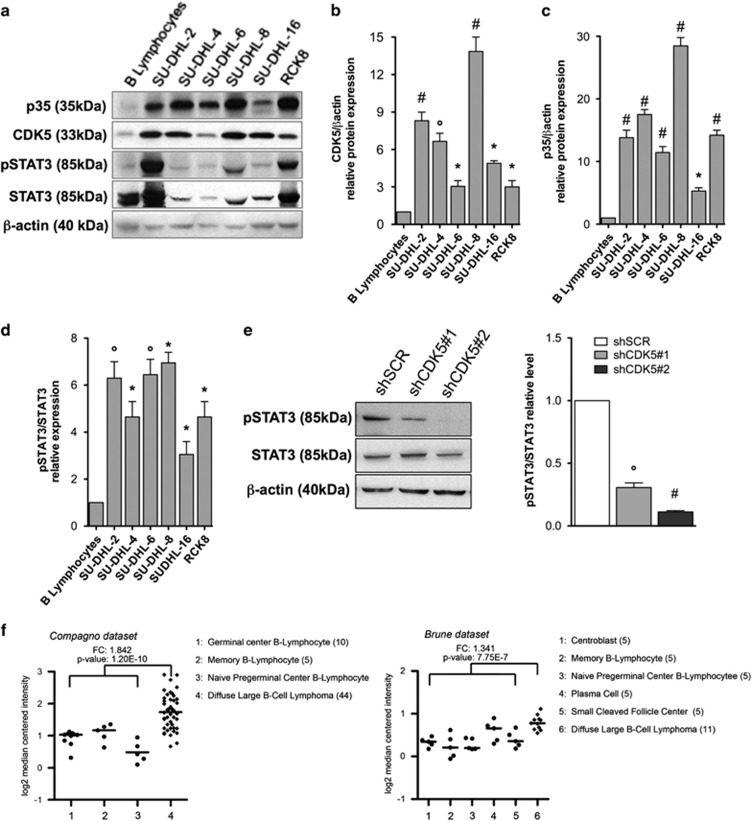
CDK5 and p35 are overexpressed in DLBCL cell lines and cancerous tissues. (**a**) Immunoblots of CDK5, p35, total STAT3 and phosphorylated STAT3 on S727 on B lymphocytes derived from human blood sample and DLBCL lymphoma cell lines. *β*-Actin level was used as loading control. (**b**–**d**) Densitometric analysis of normalized CDK5 and p35 protein to actin are presented as well as pSTAT3 refered to total STAT3. The value in the control sample (B-lymphocytes) was arbitrarily defined as 1. (**e**) Immunoblots of STAT3 phosphorylation levels (S727), total STAT3 and *β*-actin in CDK5-specific shRNAs transduced DLBCL cells. The value in the control sample (shSCR) was arbitrarily defined as 1. (**f**) Gene expression analysis of CDK5 in primary DLBCL samples (GSE_12195 and GSE_12453). Straight bars represent the median. Results are representative of minimum 3 independent experiments. The data are presented as the means±S.D. Significant differences are indicated by: ^#^*P*<0.001, *P<0.01, and *P*<0.05

Previous reports have shown that several solid tumors present an overactivation of CDK5 that correlates with the progression of the disease.^3^ Thus, we speculated that CDK5 could play a role in hematopoietic malignancies. By immunoblotting, we observed that CDK5 and its activator p35 are overexpressed in a panel of lymphoma cell lines, including DLBCL (Figures 1a–c), Hodgkin lymphoma, Burkitt lymphoma, and chronic lymphocytic leukemia cell lines (Supplementary Figure S1a) compared to B lymphocytes. In general, while a correlation exists between CDK5 mRNA and protein levels (Supplementary Figure S1b), this does not hold for p35, suggesting a different regulation of p35 protein translation between cancer and normal cells (Supplementary Figure S1c). We then focused on DLBCL and measured the level of STAT3 phosphorylation, a target of CDK5,^16^ at serine 727 (S727) in samples of DLBCL cells: as expected, DLBCL cell lines showed a higher level of phosphorylation compared to B lymphocytes (Figures 1a and d). Furthermore, using CDK5-specific shRNAs to inhibit CDK5 expression in the SU-DHL-8 cell line, we confirmed that the S727 phosphorylation of STAT3 was dependent on the expression of CDK5 (Figure 1e). We then examined the expression of CDK5 and p35 mRNA levels in DLBCL patients and healthy individuals using the published gene expression data sets. CDK5 RNA is significantly upregulated in samples of DLBCL patients compared with the relative normal counterpart from healthy individuals (Figure 1f and Supplementary Figure S1d). As expected, considering the results we obtained in DLBCL cell lines, there was no difference in p35 mRNA (Supplementary Figure S1e). Overall, these results indicate that CDK5 is overexpressed in DLBCL.

### CDK5 down-regulation in DLBCL cell lines impairs proliferation *in vitro*

**Figure 2:**
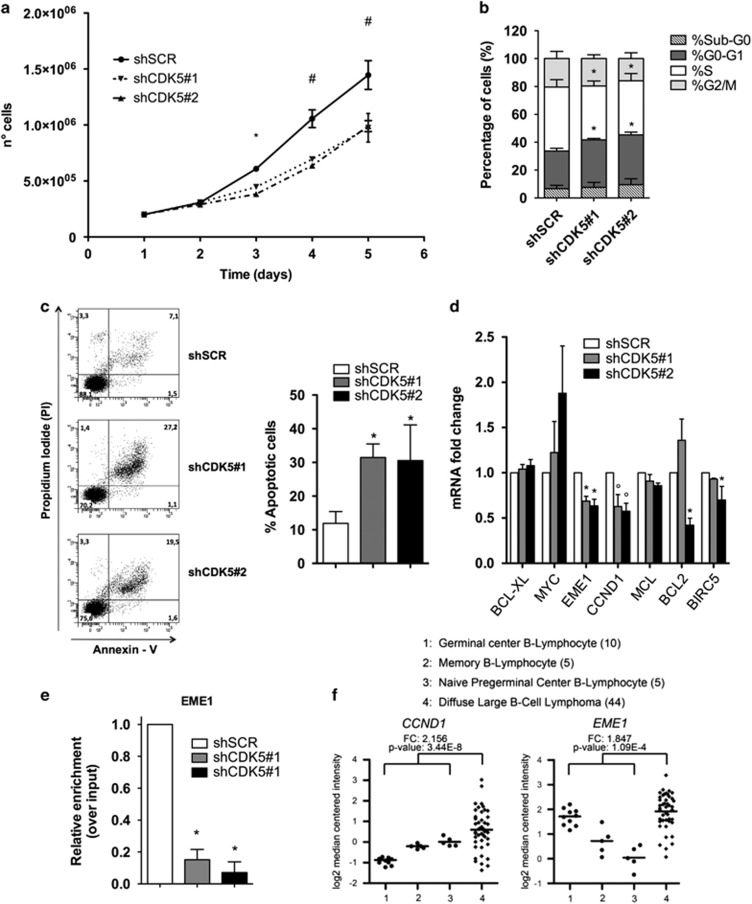
CDK5 inhibition reduces proliferation and increases apoptosis rate of DLBCL cell lines. (**a**) Cell growth curves showing decreased proliferation in CDK5-specific shRNAs transduced SU-DHL-8 cell line. (**b**) Cell cycle analysis of CDK5-specific shRNAs transduced SU-DHL-8 cell line. (**c**) Cell death rate of SU-DHL-8 cells transduced with control or CDK5-specific shRNAs was assessed by Annexin-V/PI staining. (**d**) mRNA levels of STAT3 transcriptional targets in CDK5-specific shRNAs detected by quantitative real-time PCR are shown as fold changes relative to the levels of control group (shSCR). (**e**) The occupancy of STAT3 at the EME1 promoter in CDK5-specific shRNA was analysed by ChIP assay. Relative enrichment is normalized over input DNA. (**f**) Gene expression analysis of CCND1 and EME1 in primary DLBCL samples (GSE 12195). Straight bars represent the median. Results are representative of minimum 3 independent experiments. The data are presented as the means±S.D. Significant differences are indicated by: ^#^*P*<0.001, *P<0.01, and *P*<0.05

To dissect the functional relevance of CDK5 expression and activity in modulating DLBCL malignant phenotypes, we studied the effects of CDK5 and p35 loss- and gain-of-function on proliferation in different DLBCL cell lines. We knocked down CDK5 and p35 using two different lentiviral shRNAs for each gene. In these experiments, we used the SU-DHL-8 ABC DLBCL cell line, the SU-DHL-4 and the SU-DHL-6, both GCB DLBCL cell lines, all expressing higher levels of CDK5 and p35 comparing to B-lymphocytes. In SU-DHL-8, the silencing efficiency was confirmed by qRT-PCR and western blot (Supplementary Figure S2a, S2b, S2c and S2d) and the cell growth was monitored at various time points (24, 48, 72, and 96 h). Proliferation of SU-DHL-8 cells transduced with CDK5-specific shRNAs (shCDK5#1 and shCDK5#2) was significantly inhibited (*P*<0.001) relative to control group where SU-DHL-8 cells were transduced with shRNA control vectors (shSCR) (Figure 2a), and was associated with G1 phase cell cycle arrest (Figure 2b). BrdU incorporation and MTT assays confirmed the growth curve results (Supplementary Figure S2e and S2f), suggesting that CDK5 could promote the proliferation in DLBCL cells. As expected, stable expression of p35-specific shRNAs in SU-DHL-8 inhibited significantly cell proliferation as well (Supplementary Figure S2g). Similar effects on cell proliferation were observed using CDK5-specific shRNAs in SU-DHL-4 cells (Supplementary Figure S3a and S3b) and in SU-DHL-6 cell line (Supplementary Figure S3c and S3d). To further strength these results, we performed gain-of-function experiments expressing ectopic p35 in the SU-DHL-16 cell line, which showed the lowest level of p35 expression (Figure 1a) and CDK5 activity (Figure 1c). Ectopic expression level was confirmed by western blotting compared to SU-DHL-16 cell line transduced with the empty vector (Supplementary Figure S4a). Consistent with previous results, stable expression of recombinant p35 increased cell proliferation by 37% (*P*<0.001) (Supplementary Figure S4b).

### CDK5 activity is required to protect lymphoma cells from apoptosis

Since CDK5 enhances cell survival in neurons^17^ and in podocytes,^18^ we investigated whether a similar effect could be observed in lymphoma cells. Indeed, we found that the percentage of apoptotic SU-DHL-8 cells expressing CDK5-specific shRNAs was significantly different compared to those expressing a scrambled sequence (Figure 2c). Then we evaluated whether p35 silencing might also trigger apoptosis, and we found that p35-knockdown in SU-DHL-8 cells resulted in a significantly higher cytotoxicity compared to scrambled-transduced cells (Supplementary Figure S5a). Pan-Cdk’s inhibitors (PurvalanolA and CGP 74514A) have a similar effect on apoptosis of SU-DHL-8 (Supplementary Figure S5b). On the other hand, even though SU-DHL-4 cells expressing CDK5-specific shRNA are more apoptotic, the percentage of apoptotic cells was not statistically different between SU-DHL-4 cells expressing CDK5-specific shRNAs and those expressing a scrambled sequence. The reduced level of silencing achieved in this cell line (< 50%) could probably be the reason. Thereby, we wondered whether this level of CDK5 silencing was sufficient to enhance sensitivity to the pro-apoptotic agent TRAIL in SU-DHL-4 cells, and found that CDK5-knockdown in SU-DHL-4 cells resulted in a significantly higher cytotoxicity compared to scrambled-transduced cells (Supplementary Figure S6a). Moreover, to test the hypothesis that CDK5-inhibition might be used in combination with cytotoxic agents to increase the cellular apoptotic rate, we inhibited CDK5 activity in SU-DHL-8 cells using CDK’s PAN-inhibitors in combination with the pro-apoptotic agent TRAIL (Supplementary Figure S6b and S6c).

To further demonstrate that CDK5 activity is significant to protect cells from apoptosis, we measured the apoptosis level in SU-DHL-16 cells overexpressing p35 triggered with bortezomib (BTZ) (SU-DHL-16 were not sensitive to TRAIL in our hands). Consistent with previous results, we detected a substantial decrease in the BTZ-induced apoptosis after p35 overexpression (Supplementary Figure S6d). These results strongly suggest that CDK5 activity is able to prevent apoptosis in DLBCL cells.

### CDK5 activity regulates the transcription of specific STAT3 targets

In order to investigate the molecular pathways involved in the regulation of proliferation and apoptosis driven by CDK5, we measured the expression of STAT3-specific targets in lymphoma cells expressing CDK5-specific shRNAs. Expression of two out of six genes resulted dependent on the presence of CDK5: essential meiotic structure-specific endonuclease 1 (EME1) and cyclin D1 (CYCD1) (Figure 2d). As proof of principle, we tested whether STAT3 binds to the promoter of EME1 in SU-DHL-8 cells expressing CDK5-specific shRNAs as compared to those expressing a scrambled sequence by chromatin precipitation assay (Figure 2e). This result suggests that CDK5 is able to regulate the transcriptional activity of STAT3 only on specific target genes. Interestingly, the STAT3 targets EME1 and CYCD1 also are significantly upregulated in the DLBCL patients were CDK5 resulted overexpressed (Figure 2f).

### CDK5 modulates *in vivo* tumor growth of DLBCL cell lines

**Figure 3:**
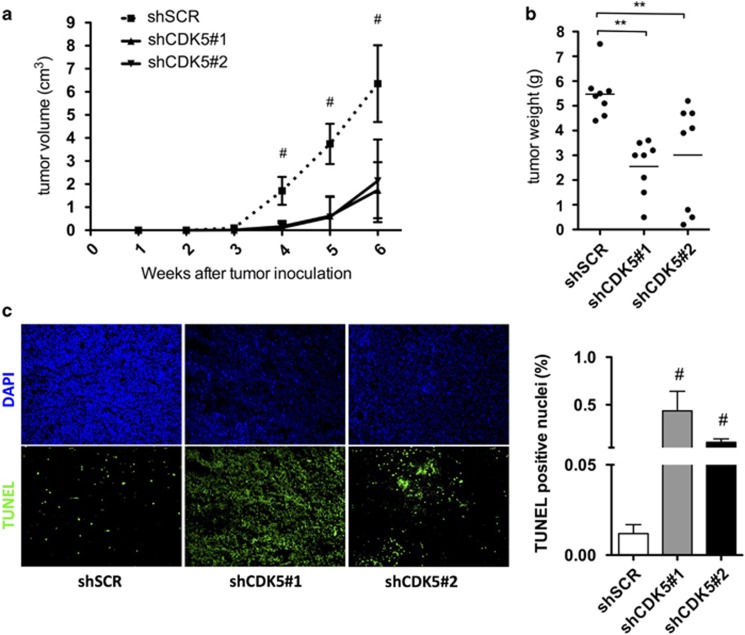
CDK5 inhibition reduces tumor growth and and increases apoptosis rate *in vivo*. (**a**,**b**) Growth curves and tumor weight (at 4 weeks post-inoculation) of xenograft tumors expressing scramble sequence (*n*=8) or CDK5-specific shRNAs (*n*=8 for both shRNAs) are shown. (**c**) TUNEL staining was used for detecting apoptosis rate induced by CDK5-specific shRNA in the xenograft model. Results are representative of minimum 3 independent experiments. The data are presented as the means±S.D. Significant differences are indicated by: ^#^*P*<0.001, *P<0.01, and *P*<0.05

To further corroborate our results, SUDHL-8 expressing CDK5-specific shRNA (shCDK5#1 and shCDK5#2), or control shRNA (shSCR) were injected subcutaneously into nude mice. Palpable tumors formed between 2–3 weeks. Tumor volume was measured every other day, and mice were killed 5 weeks after tumor cell implantation. The tumors of the SU-DHL-8 shCDK5#1 and shCDK5#2 group were not detectable for almost the entire study, while SU-DHL-8 (shSCR) presented more prominent tumors with similar average tumor volumes (Figures 3a and b). To assess tumor proliferation relative to CDK5 expression, we performed immunohistochemical analysis for Ki-67, which identifies proliferating cells, on the tumor xenografts, but we could not measure any significant difference (data not showed). The amount of apoptosis among the tumor samples was assessed by TUNEL assay. The number of apoptotic cells per field was significantly higher in tumors with defective CDK5 expression (Figure 3c). These results clearly demonstrate that CDK5 regulates tumor growth and apoptosis of DLBCL cells *in vivo*.

### MiR-26a represses p35 expression

**Figure 4:**
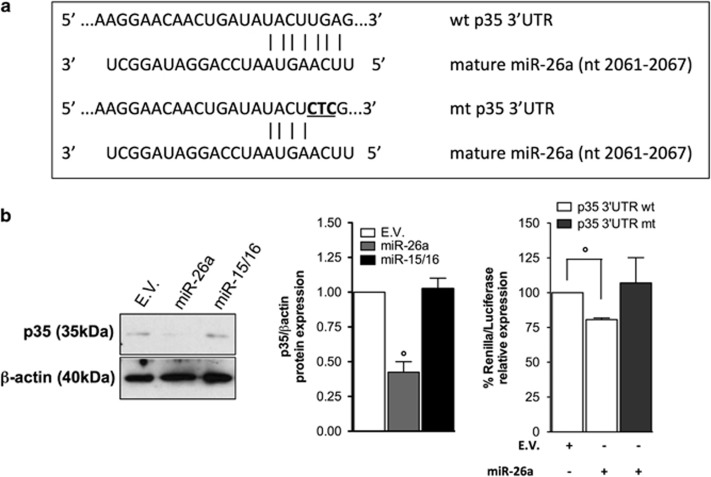
P35 is a direct target of miR-26a. (**a**) MiR-26a and its putative binding sequence in the 3′-UTR of p35. Underlined, the mutant seed sequence (wt, wild type; mt, mutant type). (**b**) Effects of hsa-miR-26a and -15/15 overexpression on native p35 protein levels in in human SU-DHL-8 lymphoma cell line with respect to the nontargeting control assessed by Western blot. *β*-Actin levels were used as loading control. Densitometric analysis of normalized p35 protein to *β*-Actin is presented. The value in the control sample was arbitrarily defined as 1. Results are representative of 2 independent experiments. (**c**) Luminescence intensity from luciferase activity of reporter plasmid with the wt or mt 3′-UTR of p35 fused to the luciferase gene following hsa-miR26a and scrambled miRNA (scrMiRNA) as nontargeting control cotransfection in HEK293T cells. The white columns represent cotransfection of the reporter plasmid with the wild-type 3′-UTR of p35 with control miRNA or cotransfection of the same reporter vector with hsa-miR-26a, while the black column the cotransfection of reporter plasmid with the 3′-UTR of p35 mutated in the putative binding site of miR-26a (C upper panel) with the hsa-miR-26a. Values are normalized to the value of control, which is noted as 100%. **Significant difference (*P*<0.01). Error bars represent SEM. Result are representative of 3 independent experiments. The data are presented as the means±S.D. Significant differences are indicated by: ^#^*P*<0.001, *P<0.01, and *P*<0.05

Since cancer cells showed higher p35 protein levels but not increased mRNA levels compared to B lymphocytes (Figure 1a and Supplementary Figure S1b), we searched for miRNAs that can potentially bind to its 3′-UTR. Then, we surveyed the literature selecting those previously described as onco-suppressors in other cancer types. This workflow suggested miR-26a as potential regulators of p35, having one putative binding site in its 3′-UTR with a seed sequence located at positions 2061–2067 bp (Figure 4a). To test whether p35 could be regulated by miR-26a, its precursor (hsa-pre-miR-26a) was expressed into SU-DHL-8 cells. Western blotting of whole cell lysates from cells overexpressing hsa-miR-26a showed a decrease of the native p35 protein (Figure 4b) compared to a scrambled miRNA. Ectopic expression efficacy was confirmed by qPCR by measuring the level of the mature miRNA (Supplementary Figure S7). To further confirm that the effect of miR-26a on p35 expression depends upon binding to its 3′-UTR, we performed a luciferase assay by fusing the p35 3′-UTR sequence to a luciferase reporter gene. By co-transfecting the hsa-miR-26a with this vector, we demonstrated that miR-26a significantly repressed luciferase activity compared to a nontargeting control. Mutagenesis of the seed sequence led to a recovery of the luciferase activity (Figure 4c). Taken together, these results indicate that p35 is a direct target of miR-26a in DLBCL cells.

### MiR-26a modulates proliferation and cell cycle progression of DLBCL cells through p35 regulation

**Figure 5:**
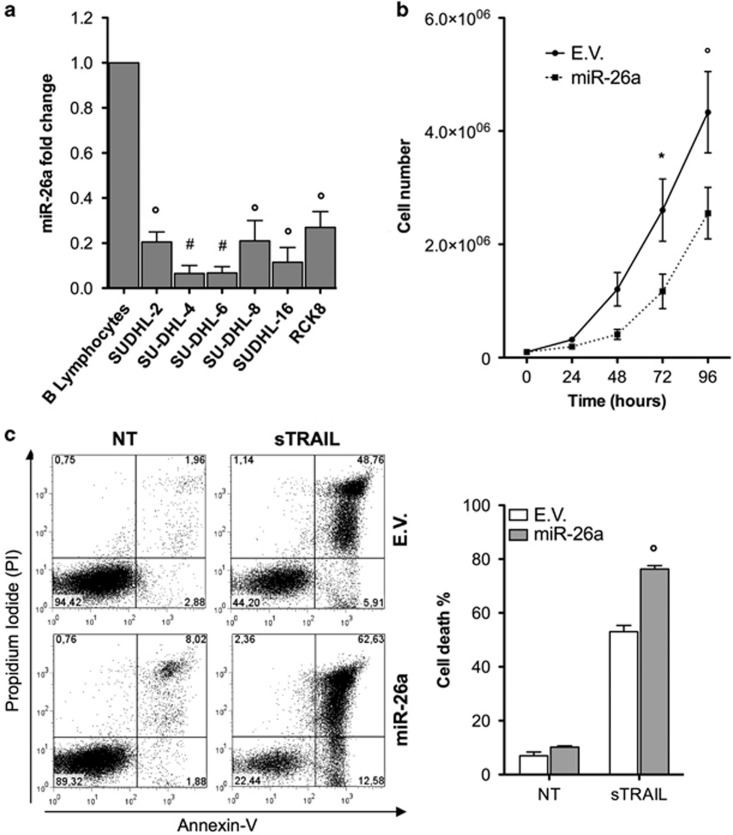
miR-26a affects proliferation and apoptosis in DLBCL cell lines. (**a**) Expression levels of miR-26a in DLBCL cell lines. (**b**) Cell growth curves showing decreased proliferation in miR-26a overexpressing SU-DHL-8 cell line. (**c**) Cell death rate of miR-26a overexpressing SU-DHL-8 was assessed by Annexin-V/PI staining before and after 48 h of sTRAIL treatment. Results are representative of minimum 3 independent experiments. The data are presented as the means±S.D. Significant differences are indicated by: ^#^*P*<0.001, *P<0.01, and *P*<0.05

**Figure 6:**
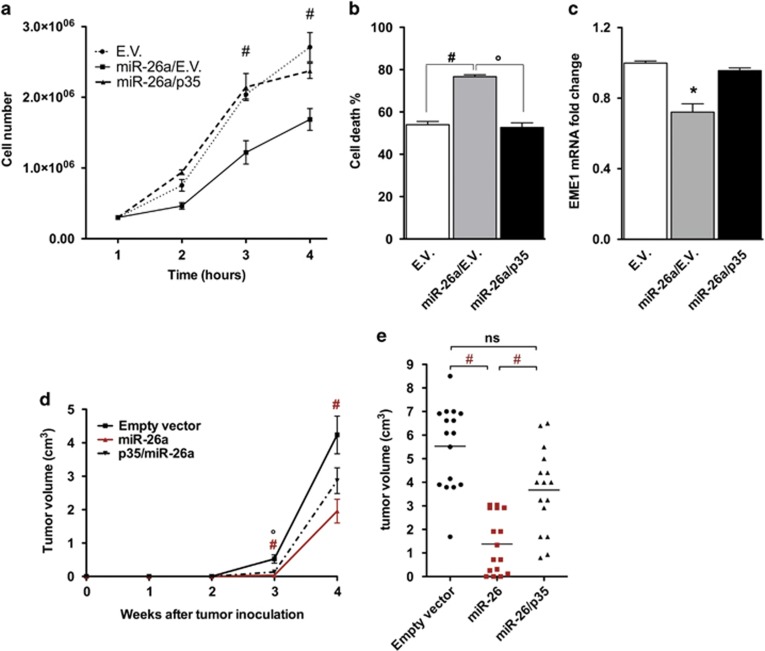
miR-26a effect on proliferation and apoptosis is rescued by overexpressing p35. (**a**,**b**) Concomitant expression of a p35 with a 3′-UTR truncated form together with miR-26a increased cell proliferation and decreased the apoptotis compared with miR-26a alone. (**c**) mRNA levels of EME1 detected by quantitative real-time PCR is rescued by overexpressing both p35 and miR-26a compared with miR-26 alone. (**d** and **e**) Growth curves and tumor weight (at 4 weeks post-inoculation) of xenograft tumors expressing scramble sequence (*n*=15), miR-26 (*n*=15) or miR26/p35 (*n*=15) are shown. Results are representative of minimum three independent experiments. The data are presented as the means±S.D. Significant differences are indicated by: ^#^*P*<0.001, *P<0.01, and *P*<0.05

First, in order to demonstrate whether miR-26 might have a role on lymphoma biology, the expression level of miR-26a was measured in DLBCL cell lines by qRT-PCR. MiR-26a was strongly downregulated in DLBCL cell lines compared to B lymphocytes (Figure 5a). Next, we investigated the role of miR-26a in lymphoma cells. To this end, using a lentiviral system, we examined the effects of the hsa-miR-26a overexpression on the proliferation rate of SU-DHL-8 ABC cells. Compared to controls, hsa-miR-26a overexpression decreased cell proliferation by 41% (*P*<0.01) (Figure 5b). Moreover, hsa-miR-26a expression substantially augmented sTRAIL–induced apoptosis in SU-DHL-8 cells, while the induction of apoptosis in untreated cells was not statistically significant (Figure 5c). In addition, hsa-miR-26a stable expression significantly increased BTZ-induced apoptosis not only of SU-DHL-8 cells but also in the previously described BTZ-resistant cells SU-DHL-4^19^ and SU-DHL-6^20^ (Supplementary Figure S8). Then, in order to demonstrate that the effect of miR-26a on proliferation and apoptosis of DLBCL was mediated by p35 and consequently modulation of CDK5 activity, we generated new cell lines: starting from SU-DHL-8, we created a control line (E.V.), a cell line overexpressing miR-26a (miR-26/E.V.), and a cell line overexpressing miR-26a together with a vector expressing the human p35 ORF and missing the 3′-UTR (miR-26/p35). Modulation of p35 expression and miR-26a overexpression was confirmed by qPCR (Supplementary Figure S9). Compared to miR-26/E.V. cells, miR-26/p35 clone markedly resulted in an increase in cell proliferation and a decrease in apoptosis, similar to the proliferation and apoptosis index detected in E.V. cells (Figures 6a and b). These data demonstrate that miR-26a regulates proliferation and apoptotic events in DLBCL cells and that p35 is an important mediator of these biological events. In order to explore whether the miR-26a/CDK5/p35 axis exerts its functions through the CDK5/STAT3 pathway, we then examined the expression level of the different STAT3 target gene that was significantly downregulated upon CDK5 silencing EME1. Expression levels of EME1 was decreased in SU-DHL-8 cells expressing miR-26/E.V., but not in SU-DHL-8 cells expressing miR-26/p35 compared to E.V. control cells (Figure 6c). These data indicate that miR-26a inhibits CDK5/STAT3 signaling in DLBCL cells.

To further strength these results, SU-DHL-8 cells stably expressing miR-26a (miR-26/E.V.), cells overexpressing miR-26a together with a vector expressing the human p35 and missing the 3′-UTR (miR-26/p35), or control vectors (E.V.) were injected subcutaneously into nude mice. The tumors of the SU-DHL-8 miR-26/E.V. group were significantly smaller than SU-DHL-8 (E.V.) for almost the entire study, while SU-DHL-8 (miR-26/p35) lost the difference in tumor growth compared to the control (Figures 6d and e).

## Discussion

Since its discover, CDK5 has been manly studied for its role in the regulation of neuronal cell biology. However, recently several groups have identified a link between CDK5 and human cancers: CDK5 activity in cancer cell migration and invasion, and consequently, in tumor progression has been well documented in different solid tumors, such as breast,^21^ prostate,^4^ lung,^22^ and pancreatic^5^ cancers. Nonetheless, few studies investigated CDK5’s role in hematopoietic malignancies, such as leukemia^23^ and multiple myeloma,^24^ while none has evaluated its potential role in DLBCL. In this study, we showed for the first time that human DLBCL cells present an increased expression of CDK5 at both protein and mRNA levels compared to normal lymphocytes, and these data were also confirmed in samples derived from patients with DLBCL as compared to normal B cells. This particular modulation of CDK5 in DLBCL cells might depend by epigenetic modifications; indeed, in a previous study, Leshchenko and colleagues demonstrated that CDK5 is one of the most hypomethylated genes in mantle cell lymphoma.^25^

P35, a critical regulator of CDK5 activity, is a short-lived protein and its expression is finely regulated through ubiquitin-proteasome degradation^26^ or through the presence of regulatory elements affecting its transcript stability.^27^ We observed that, while p35 is virtually absent in healthy controls, it results to be upregulated in cancer cells at protein but not at RNA level. In order to define how p35 might be regulated in DBCL, we looked for additional post-transcriptional regulatory process able to inhibit the translation of p35 in normal cells, but absent on tumor cells. Thereby, we thought that a mechanism involving microRNAs, that have been associated with tumorigenesis,^9^ might be the answer. For instance, deregulation of miR-26a expression has been correlated with cancer progression, whereas it is still not clear whether it might act as a tumor suppressor or as an oncogene.^10, 11, 12, 13, 14, 15^

Here, we first demonstrate that DLBCL cells express low levels of miR-26a compared to non-tumor cells, then we found that miR-26a directly affects p35 expression in DLBCL cells, and that their amounts are inversely correlated in cancerous cells. However, the specific regulation of miR-26 in DBCL is not clear, thereby we can speculate that an epigenetic mechanism might be involved, as previously observed for other microRNAs associated to cancer development.^28^ Then, we showed that the up-regulation of miR-26a significantly reduces p35 levels in DLBCL cells and that its overexpression decreases the luciferase reporter activity of a plasmid carrying a p35 wild-type 3′-UTR, while this reduction is blunted when the miR-26a seed sequence is mutated. In addition, in the attempt to clarify the function of miR-26a/CDK5/p35 axis in DLBCL, we silenced CDK5 or p35 and observed a significant decrease in DLBCL cell proliferation. Moreover, miR-26a overexpression suppresses cell proliferation of DLBCL cells *in vitro*, and *in vivo* inhibits DLBCL tumor growth at least in part by suppressing p35. The effect of miR-26a modulation on cell proliferation and tumor growth of DLBCL cells was accompanied by changes in p35 levels and CDK5 activity. Furthermore, the concomitant expression of a recombinant p35 lacking of the 3′-UTR completely abrogates the effects induced by miR-26a. All together, these results clearly indicate that miR-26a acts as a tumor suppressor in DLBCL cells, and this might depend by the regulation of different genes, including p35.

Resistance to apoptosis is a hallmark of cancer and the attenuation of such capacity might be a valuable anticancer therapy strategy.^29^ For instance, tumors often increase the expression of anti-apoptotic regulators, such as Bcl-2 and related protein family, and inhibit the expression of pro-apoptotic factors, such as Bax, and caspase-3. Therefore, the identification of new mechanisms underlying apoptotic pathways is of great importance in order to identify alternative strategy to treat cancer. The present study demonstrated that the miR26/CDK5 axis is important in order to promote an anti-apoptotic environment for DLBCL cells. The increased expression of p35 in DLBCL cells enhances the resistance to apoptosis induced by BTZ (the first proteasome inhibitor utilized as chemotherapeutic drug for the treatment of several types of cancers). By contrast, the knockdown of CDK5/p35 or overexpression of miR-26a markedly decreases the ability of DLBCL cells to resist to apoptosis.

The function of CDK5 in DLBCL might be explained also by taking into account the cellular role of previously identified CDK5 targets. For instance, CDK5 phosphorylates Ataxia telangiectasia mutated (ATM) and, by mediating its activation, regulates DNA repair.^30^ In response to DNA damage and through the CDK5/ATM signaling, p53 activates the expression of some important target genes related to cell death, including PUMA and BAX.^31^ In addition, Courapied and colleagues showed that, upon DNA damage, CDK5 phosphorylates STAT3 on S727 and activates the transcription of EME1, an endonuclease involved in DNA repair.^32^ Moreover, it has been demonstrated that STAT3 is a master regulator of tumorigenesis, by modulating the expression of many survival genes.^33^ Using RT-qPCR, we showed that overexpression of miR-26a leads to a significant decrease of the EME1 mRNA level, while the concomitant expression of a 3′-UTR-truncated form of p35 restores the expression of this gene to near basal levels; this suggests that in DLBCL cells the STAT3 pathway is one of the most important mediators of CDK5 activity.

All together, these findings support the notion that the CDK5/p35/STAT3 pathway mediates the tumor-suppressive function of miR-26a, and that p35 deregulation through miR-26a plays an important role in tumor growth. However, miR-26a on one hand and p35 on the other might respectively regulate or be regulated by other genes as well, thereby further studies might be needed in order to finally evaluate their potentiality as novel prognostic markers and/or potential therapeutic targets for DLBCL.

## Materials and methods

### Cell lines

DLBCL cell lines used in this study included Germinal Center B-cell (GCB) cell lines (SU-DHL-4, SU-DHL-6, SU-DHL-16) and activated B-cell (ABC) cell lines (SU-DHL-2, SU-DHL-8, and RCK-8), and were kindly provided by L Pasqualucci, M.D. (Columbia University, New York, NY, USA). Cell lines were cultured in RPMI-1640 (Lonza, Walkersville, MD, USA) supplemented with 10% fetal bovine serum (FBS, Sigma-Aldrich, St Louis, MO, USA), 10 000 U /ml penicillin, 10 000 mg/ml streptomycin (Pen/Strep, Lonza), 20 mM L-Glutamine (Ultraglutamine, Lonza), and periodically tested for mycoplasma contamination. Normal B lymphocytes were negatively selected by an immunomagnetic technique (Human B Cell Enrichment Kit, Stem Cell Technologies, Vancouver, BC, Canada) from the peripheral blood of consenting healthy donors.

### Generation of loss- and gain-of-function of CDK5/p35 or miR-26a expression on stable DLBCL cell lines

Lentiviral CDK5 and p35 short hairpin RNA (shRNA) (shCdk5#1 and #2, shp35#1 and #2) were obtained from the RNAi Consortium (http://www.broadinstitute.org/rnai/trc). A scramble shRNA was used as a control. Lentiviral particles were generated using a three-plasmid system, as described previously.^34^ For p35-expressing lentiviral particles, a p35-expressing vector containing the human CDS p35 complementary DNA (NM_003885.2) was cloned in lentiviral plasmids. DLBCL cells were transduced with lentiviral particles and polybrene at 8 *μ*g/ml (Sigma-Aldrich), followed by puromycin selection at 48 h after the transduction. Efficiency of knockdown or overexpression was validated by immunoblotting and/or qRT-PCR.

### Proliferation assay

For growth curves, all cell lines were plated and grown under normal conditions for 1–4 days. Every day, cells were collected, diluted in Trypan blue to assess viability, and counted in the Burker’s chamber. Cell proliferation was further assessed by MTT assay according to the manufacturer's recommendations (Roche Diagnostics GmbH, Mannheim, Germany): 40 000 cells were seeded in triplicates in a 96-wells; 5 mg/ml MTT per well was added both 24 and 48 h after seeding. Cells were then incubated for 4 h and DMSO was used to dissolve crystals. 5-Bromo-2'-Deoxyuridine (BrdU) incorporation was also used to confirm cellular proliferation. Cells were plated and grown under normal conditions for 24 h. Cells were labeled with 3 *μ*g/ml BrdU for 4 h, washed twice with PBS, fixed in 1% formaldehyde and then spotted on slides. immunofluorescence was performed using anti-BrdU antibody (Santa Cruz Biotechnology, Santa Cruz, CA, USA), according to the manufacturer’s protocol.

### Apoptosis assays

Apoptotic cell death was detected by Annexin-V–Allophycocyanin (APC)/Propidium Iodide (PI) double staining (Immunostep, Salamanca, SP, EU), according to the manufacturer’s instructions. This staining allows quantification of early (annexin-V^+^/PI^−^) and late (annexin-V^+^/PI^+^) apoptotic cells, as well as necrotic cells (annexin-V^−^/PI^+^). In order to detect cell death, cells were recovered after 24 h, double stained with Annexin-V and propidium iodide and analyzed on a FACSCalibur flow cytometry system (Becton-Dickinson, San Jose, CA, USA) using BD CellQuest software version 3.3 (Becton-Dickinson, San Jose, CA, USA). Data were analyzed using FlowJo 7.3.5 software for Windows (Tree Star, Inc. Ashland, OR, USA).

### Cell cycle analysis

Cells (1 × 10^6^) were washed twice with PBS and fixed in 70% ethanol and kept at 4 °C prior to overnight DNA staining with 2.5 *μ*g/ml PI (Calbiochem, Darmstadt, Germany) in the presence of 12.5 *μ*g/ml RNAse (Sigma-tau, Rome, Italy). The number of cells at each stage of the cell cycle was measured using a FACSCalibur flow cytometry system (Becton-Dickinson). The histograms were analyzed using the FlowJo 7.3.5 software version for Windows (Tree Star).

### Western blot analysis

Cell samples were homogenized in NP-40 lysis buffer (1% NP-40, 20 mM Tris-HCl pH 8, 137 mM NaCl, 10% glycerol, 2 mM EDTA, 1 mM sodium orthovanadate, 10 *μ*g/ml aprotinin, 10 *μ*g/ml leupeptin). Protein concentrations were determined by using bicinchoninic acid (BCA) Protein Assay. Cell lysates were resolved by electrophoresis on a 10% polyacrylamide gel and transferred to nitrocellulose membranes. Immunocomplexes were visualized using an enhanced chemiluminescence western blotting detection system (Amersham Biosciences, Milano, Italy). Blotting analysis was performed, according to the manufacturer’s protocols, using the following antibodies: CDK5 (1:1000), p35 (1:1000) (Santa Cruz Biotechnology), GAPDH (1:1000) (Cell signaling, Danvers, MA, USA), Actin (1:1000) (Sigma-Aldrich), STAT3 (1:1000) and pSTAT3 (Ser727) (1:1000) (Cell signaling). HRP- conjugated secondary antibodies (1:5000) (Pierce) against mouse or rabbit were used to detect each protein line.

### RNA extraction and real-time PCR

Total RNA was extracted using Purezol reagent (Bio-Rad, Hercules, CA, USA) following the manufacturer's instructions. Two micrograms of total RNA was used for cDNA synthesis with Oligo d(T) primer. Real-time PCR was carried out on an Applied Biosystems 7900HT Fast Real-Time PCR. PCR reactions were run in duplicate for, at least, three independent experiments. The mRNA levels were normalized to GAPDH as a housekeeping gene and analyzed using the ΔΔCt method.

### Reporter assay

For the 3′-UTR reporter assay, experiments were performed in 3T3 cells. 3′-UTR segments were sub-cloned by standard procedures into the psiCHECK-2 (Promega, Madison, WI, USA) immediately downstream of the stop codon of the renilla gene using the primers p35–5′ (5′-GAGGCTGCTTCGGATGGAGGGA-3′), p35–3′ (5′-TAAGATTTAACATCATCATATT-3′). Seed sequence mutagenesis was performed as described by the manufacturer (Agilent Technologies, Lexington, MA, USA). Luciferase assay was performed as previously described.^35^

### Chromatin immunoprecipitation assay

Cells were cross-linked for 15 min at RT using 1% formaldehyde. Cross-linking was quenched by adding glycine to a final concentration of 0.125 M. The cells were then resuspended in lysis buffer (5 mM PIPES pH 8, 85 mM KCl, 0.5% NP-40 and protease inhibitors) and incubated on ice for 15 min. Chromatin was sheared to generate 200−400 bp fragments. The efficiency of sonication was assessed with agarose gel electrophoresis. Chromatin samples were pre-cleared for 1 h with protein-G beads and then immunoprecipitated overnight at 4 °C with anti-STAT3. Immunocomplexes were washed with low-salt wash buffer (0.1% SDS, 2 mM EDTA, 20 mM Tris-HCl pH 8, 1% Triton X-100, 150 mM NaCl and protease inhibitors), high-salt wash buffer (0.1% SDS, 2 mM EDTA, 20 mM Tris-HCl pH 8, 1% Triton X-100, 500 mM NaCl and protease inhibitors) and TE buffer. Immunocomplexes were then eluted in elution buffer (1% SDS and 100 mM NaHCO3) and cross-linking reverted overnight at 65 °C. Samples were extracted with phenol/chloroform and precipitated with ethanol. Enriched regions relative to input DNA were detected by real-time PCR and analyzed using the ΔΔCt method.

### *In vivo* tumor growth of engineered SU-DHL-8 cell lines in immunodeficient (NOD/SCID) mice

Six- to eight-week-old NOD/SCID mice (Charles River Laboratories, Milano, Italy) with body weights of 20–25 g were used to generate xenografts of SU-DHL-8 cells. Experiments were performed according to Italian laws (D.L. 116/92 and following additions) and approved by the institutional Ethical Committee for Animal Experimentation. Engineered cells (10 × 10^6^ cells per mouse) were injected subcutaneously (SC) into the left flank of each mouse. The endpoint of the experiment was tumor weight. Tumors were measured with calipers, and their weights were calculated using the formula: (*a* × *b*^2^)/2, where *a* and *b* represented the longest and shortest diameters, respectively. Mice were monitored twice weekly and were sacrificed by CO_2_ inhalation when they showed signs of terminal illness, including hind leg paralysis, inability to eat or drink, and/or moribund. Each experiment was performed on at least two separate occasions, using five mice per experiment.

### Apoptosis assay *in vivo*

Sections (2 *μ*m) from formalin-fixed, paraffin-embedded tumor nodules were processed with DeadEnd Fluorometric TUNEL (Promega, Madison, WI, USA) according to the manufacturer’s instructions. The sections were examined using a light microscope (BX53; Olympus, Tokyo, Japan). Image analysis was performed using open source ImageJ software (http://rsb.info.nih.gov/ij/).

### Bioinformatics

To identify the potential miR-26a targets, the algorithms miRanda (http://www.microrna.org), TargetScan (http://www.targetscan.org), and PicTar (http://pictar.mdc-berlin.de/) were used as previously described.^36^

### Statistical analysis

Statistical analyses were performed using Prism 7.2.5 (GraphPad Software, Inc., La Jolla, CA, USA). To test the probability of significant differences between untreated and treated samples, a two-way analysis of variance was employed, and individual group comparisons were evaluated using the Bonferroni post *hoc* test. Terminal deoxynucleotidyl transferase dUTP nick end labeling and the immunohistochemistry data were analyzed using one-way analysis of variance and individual group comparisons were evaluated using the Bonferroni post *hoc* test. A two-tailed value of *P*<0.05 was considered significant. The data are represented as mean±S.D. unless otherwise stated.
