# Supplementary material for: MicroRNA-26a/cyclin-dependent kinase 5 axis controls proliferation, apoptosis and in vivo tumor growth of diffuse large B-cell lymphoma cell lines
Source: Cell Death Dis. 2017 Jun 22;8(6):e2890–. doi: 10.1038/cddis.2017.291 (PMC5520941; doi:10.1038/cddis.2017.291)
Supplement: Supplementary Material [file cddis2017291x1.pdf]

SUPPLEMENTAL MATERIAL

MicroRNA-26a/Cyclin-dependent kinase 5 axis controls proliferation, apoptosis and in vivo tumor growth of Diffuse large B-cell lymphoma cell lines

Short title: MiR-26a/CDK5 axis in Diffuse large B-cell lymphoma

Floriana Maria Farina*,[1] Alessandra Inguscio*,[1,2] Paolo Kunderfranco,[1] Alice L. Cortesi,[3] Leonardo Elia[1,4] and Manuela Quintavalle[1]

[1] Humanitas Clinical and Research Center, Rozzano (MI), Italy

[2] Department of Medical Biotechnology and Translational Medicine, University of Milano, Milano, Italy

[3]INGM, Milan, Italy

[4]Department of Molecular and Translational Medicine, University of Brescia, Italy

Correspondence: Manuela Quintavalle, Via Manzoni 113, 20089 Rozzano (MI), Italy; Telephone number: +390282245220; Email: manuela.quintavalle@humanitasresearch.it; .Leonardo Elia, Viale Europa 11, 25123 Brescia, Italy; Telephone number: +390282245211; Email: leonardo.elia@unibs.it.

* These authors contributed equally to this work

Supplemental Figures and Figure Legends

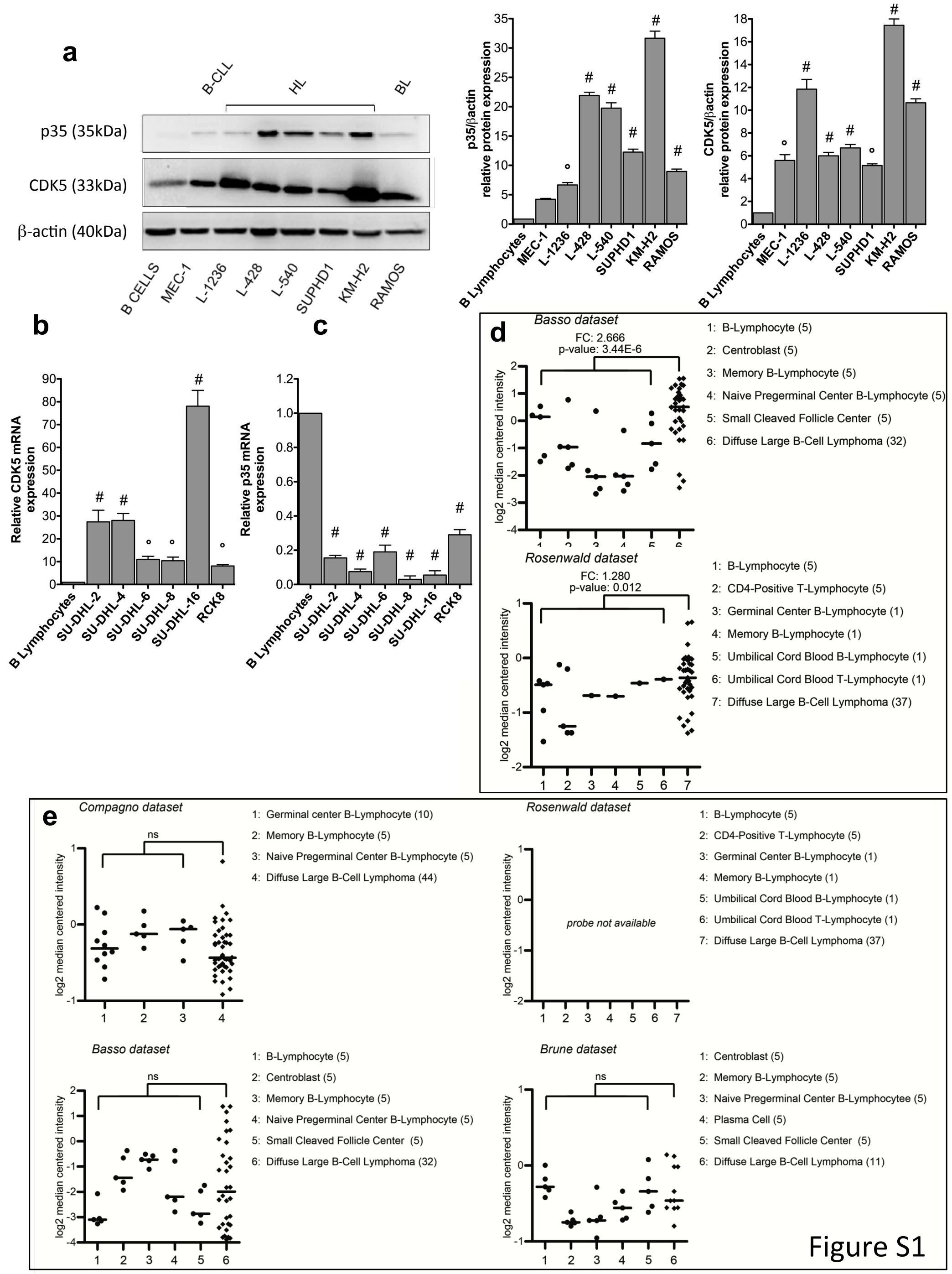

Figure S1. CDK5 and p35 are overexpressed in Lymphoma and Leukemia cell lines. (a) Western blot analysis for both CDK5 and p35 on total cell lysates from B lymphocytes, B-cell chronic lymphocytic leukemia (B-CLL), Hodgkin lymphoma (HL) and Burkitt's lymphoma (BL) cell lines. (b and c) mRNA levels of both CDK5 and p35 detected by quantitative real-time PCR in DLBCL cell lines. (d) CDK5 ene expression analysis of CDK5 in primary DLBCL samples (GSE_2350 and https://llmpp.nih.gov/DLBCL). Straight bars represent the median. (e) P35 gene

expression analysis of p35 in primary DLBCL samples (GSE_12195, https://llmpp.nih.gov/DLBCL, GSE_2350 and GSE_12453). Straight bars represent the median.
Results are representative of minimum 3 independent experiments. The data are presented as the means ± SD. Significant differences are indicated by: #P < 0.001, *P < 0.01, and °P < 0.05.

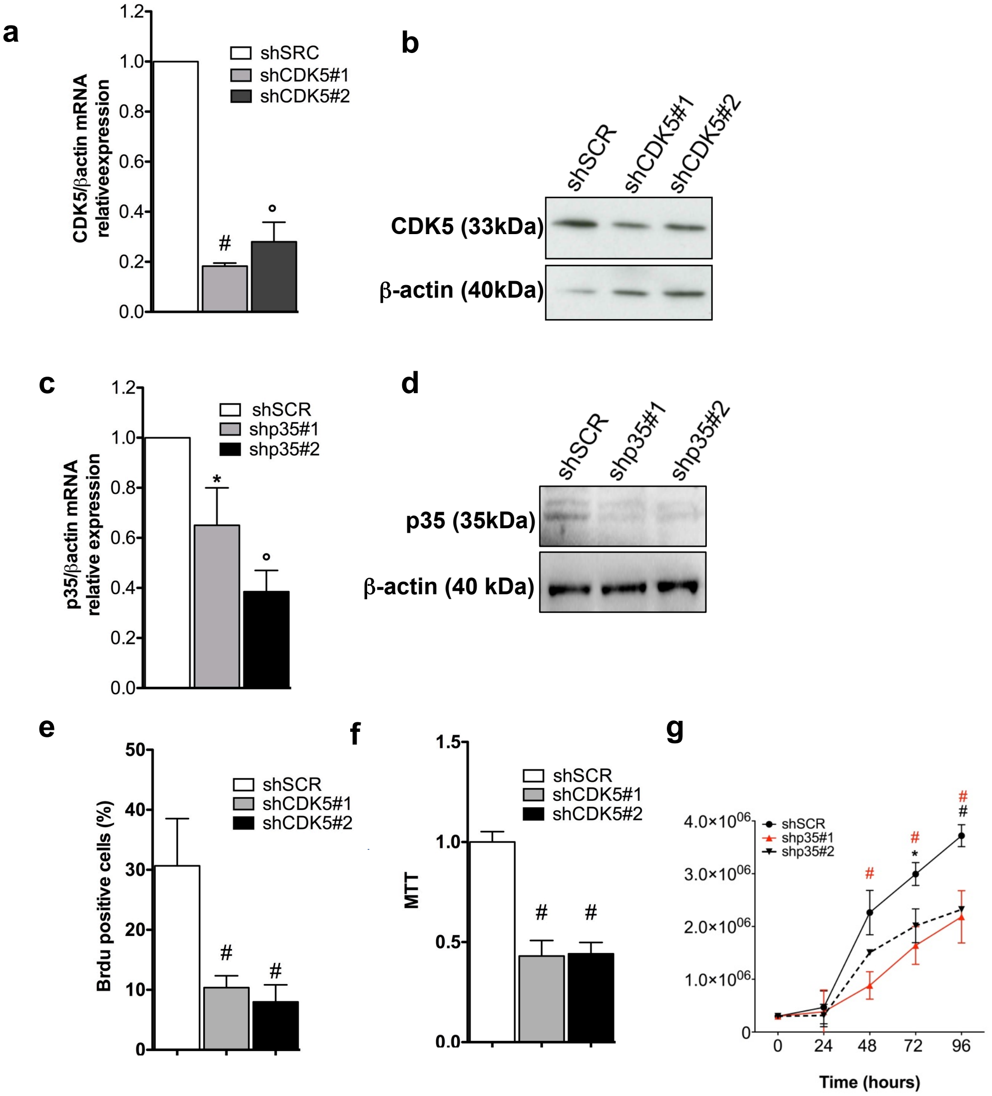

Figure S2

Figure S2. CDK5 regulates proliferation rate of DLBCL cells. Assessment of the repression efficiency of CDK5 (a and b) and p35 (c and d) expression after lentiviral infection in SUDHL8 cells. Error bars represent the mean ± SD of triplicate experiments. (e) BrdU incorporation and (f) MTT assays showing decreased proliferation rate in CDK5-specific shRNAs transduced SU-DHL-8 cell line. (g) Cell growth curves showing decreased growth of p35-specific shRNAs transduced SU-DHL-8 cell line. Results are representative of minimum 3 independent experiments. The data are presented as the means ± SD. Significant differences are indicated by: #P < 0.001, *P < 0.01, and °P < 0.05.

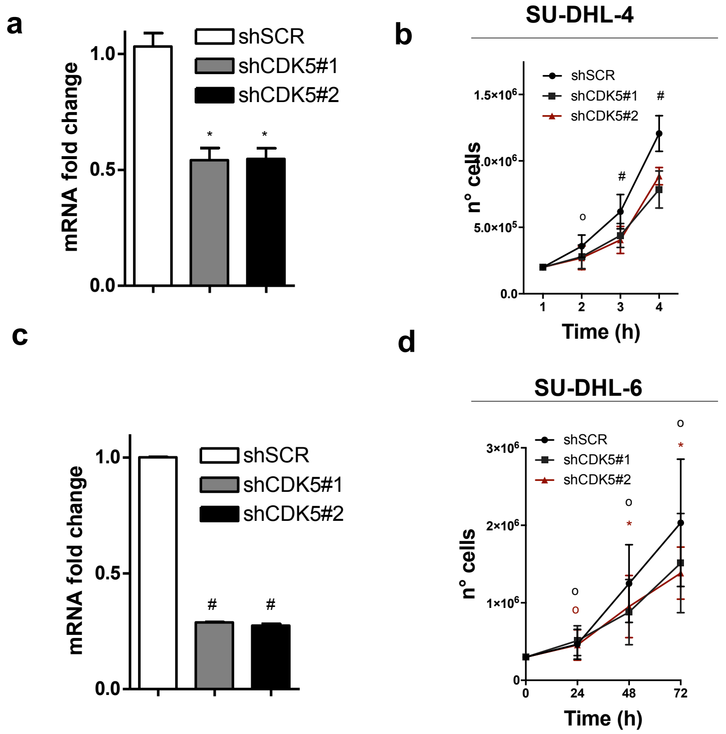

Figure S3

Figure S3. CDK5 regulates proliferation rate of GCB DLBCL cells. (a) CDK5 mRNA levels in CDK5-specific shRNA transduced SU-DHL-4 cell lines. (b) Proliferation assay in CDK5-specific or SCR shRNA transduced SU-DHL-4 cell lines. (c) CDK5 mRNA levels in CDK5-specific shRNAstransduced SU-DHL-6 cell lines. (d) Proliferation assay in CDK5-specific or SCR shRNA transduced SU-DHL-6 cell lines. Results are representative of minimum 3 independent experiments. The data are presented as the means ± SD. Significant differences are indicated by: #P < 0.001, *P < 0.01, and °P < 0.05.

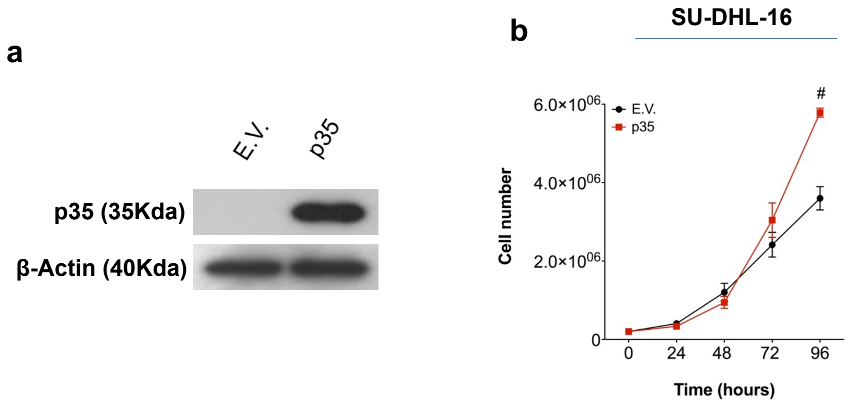

Figure S4

Figure S4. P35 ectopic expression in SU-DHL-16 increases proliferation rate. (a) p35 protein expression in SU-DHL-16 transduced with empty lentivirus vector as control and p35overexpression vector, assessed by Western Blot. (b) Cell growth of the control and of the p35 overexpression cells was monitored for 24, 48, 72 and 96 hours. Results are representative of minimum 3 independent experiments. The data are presented as the means ± SD. Significant differences are indicated by: #P < 0.001, *P < 0.01, and °P < 0.05.

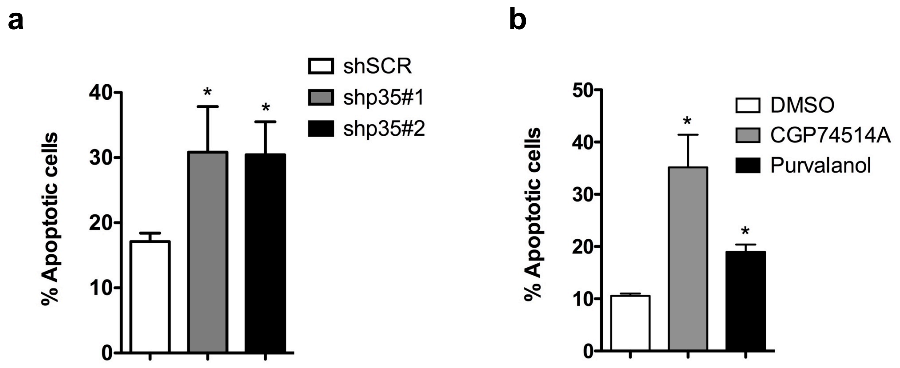

Figure S5

Figure S5. Apoptosis in DLBCL. Apoptosis in SU-DHL-8 cell lines after p35-specific shRNAs (a) and pan-CDK's inhibitors (b) treatment (CGP7414A 1μM and Purvalanol 10μM for 24h). Results are representative of minimum 3 independent experiments. The data are presented as the means ± SD. Significant differences are indicated by: #P < 0.001, *P < 0.01, and °P < 0.05.

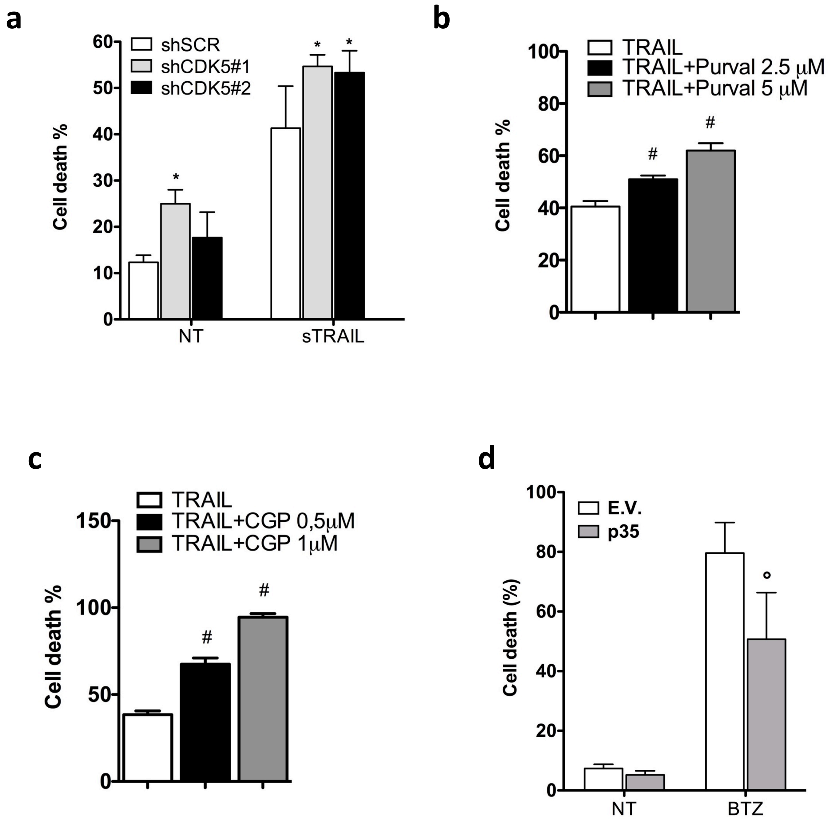

Figure S6

Figure S6. CDK5's inhibitors and specific shRNAs regulate apoptotic rate of DLBCL cells. (a) Annexin V/ PI staining was used for detecting apoptotic cell before and after sTRAIL treatment in SU-DHL-4 expressing CDK5-specific shRNAs. (b and c) Annexin V/PI staining of SU-DHL-8 sTRAIL-treated cells exposed to PURVALANOL and CGP. (d) Another pro-apoptotic agent (BORTEZOMIB) was used to assess apoptosis rate in p35 overexpressing SU-DHL-16 . Results are representative of minimum 3 independent experiments. The data are presented as the means ± SD. Significant differences are indicated by: #P < 0.001, *P < 0.01, and °P < 0.05.

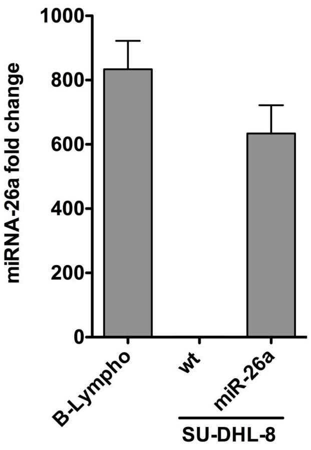

Figure S7

Figure S7. MiR-26 levels in DLBCL cells. MiR-26a was measured in wt and miR-26 over-expressing SU-DHL-8 compared to primary B-Lymphocytes. The data are presented as the means ± SD.

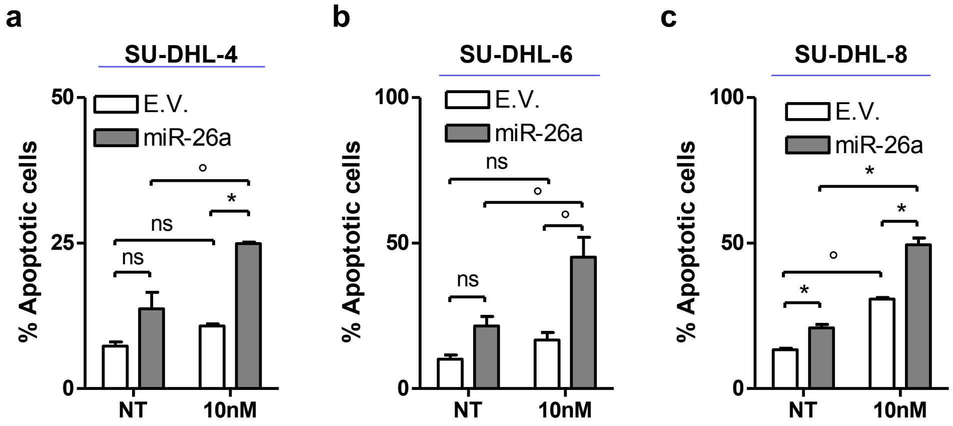

Figure S8

Figure S8. BTZ effects on SU-DHL-4 , -6 and -8 miR-26a overexpressing cells. Cells were treated for 24h with 10nM of BTX and apoptosis evaluated by Annexin V/ PI staining (a, b and c). The data are presented as the means ± SD. Significant differences are indicated by: *P < 0.01, °P < 0.05 and ns=not statistic.

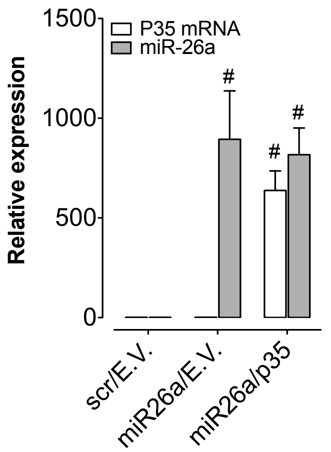

Figure S9

Figure S9. CDK5 and p35 endogenous and ectopic expression in Lymphoma cells. MiR-26a and p35 mRNA expression in DLBCL cell lines. Results are representative of minimum 3 independent experiments. The data are presented as the means ± SD. Significant differences are indicated by: #P < 0.05.
